# Unravelling the Anatomy of the Anterior Tarsal Tunnel and Its Clinical Implications

**DOI:** 10.7759/cureus.64282

**Published:** 2024-07-10

**Authors:** Jahira Banu, Nithya Dhakshnamoorthy, Sulochana Sakthivel

**Affiliations:** 1 Anatomy, Jawaharlal Institute of Postgraduate Medical Education and Research, Pondicherry, IND

**Keywords:** ankle arthroscopy, extensor digitorum longus, extensor hallucis longus, extensor retinaculum, dorsalis pedis artery, deep peroneal nerve

## Abstract

Background: Anterior tarsal tunnel (ATT) syndrome is caused by the compression of the deep fibular nerve (DFN) within the ATT beneath the inferior extensor retinaculum, bounded by the tendons of the extensor hallucis longus (EHL) and extensor digitorum longus (EDL). Compression may result from direct trauma, repetitive mechanical irritation, and thrombosis of the dorsalis pedis artery. Injury to the contents of ATT could occur during ankle arthroscopy. Therefore, this study was undertaken to provide a detailed description of the anatomy of the ATT and its clinical implications.

Materials and methods: Ten formalin-fixed cadavers were utilized for the study. The ATT was identified between the tendons of the EHL and EDL. The length at the medial and lateral boundaries and the width at the proximal end, middle, and distal end of the ATT were measured using a digital Vernier calliper.

Result: The mean length of the medial border of the tunnel was 31.42±8.44 mm, while the lateral border was 20.39±4.39 mm. The width of the ATT increased from the proximal to the distal end. DFN was related to the DPA laterally in 15 limbs and medially in five limbs within the tunnel.

Conclusion: The present study not only describes the intricate anatomy of the ATT but also describes the patterns of DFN and DPA within the tunnel. Understanding the anatomy of ATT is crucial, as it paves the way for safe and efficient surgical interventions, thereby significantly reducing the risk of neurovascular damage during surgical procedures.

## Introduction

The term "tarsal tunnel syndrome" usually refers to the involvement of the posterior tibial nerve and the structures running beneath the flexor retinaculum of the lower limb. Anterior tarsal tunnel (ATT) syndrome is a condition caused by the compression of the deep fibular nerve (DFN) as it passes beneath the inferior extensor retinaculum. Anatomically, the tarsal tunnel is divided into the anterior and posterior tarsal tunnels. The ATT is located anterior to the talotibial and talonavicular joints, whereas the posterior tarsal tunnel is located posterior to the medial malleolus of the tibia [1]. The anatomy of the ATT is described as a flattened area/space between the extensor hallucis longus (EHL) and extensor digitorum longus (EDL) tendons in the lower leg. It is bounded superficially by the inferior extensor retinaculum and deeply by the fascia covering the talus and navicular bone [2].

The contents of the ATT, the DFN, and the dorsalis pedis artery (DPA) are unprotected, as they lie superficially, making them vulnerable to injury [3]. The compression of the DFN under the inferior extensor retinaculum precipitates ATT syndrome for various reasons, such as direct trauma, extreme plantar flexion in high-heeled shoes and high boots, continuous mechanical irritation like tightly laced shoes, and thrombosis of the DPA [3-6]. The presentable symptoms might mimic lumbar radiculopathy or ankle arthritis [7, 1]. During arthroscopic surgery using the anterocentral portal, the arthroscope encountered DPA in 90% of the cases [8]. These clinical conditions show the importance of the anatomy of ATT and highlight the relevance of our research.

Even though there was uncertainty regarding the presence of this tunnel, subsequent investigations have provided clarity [3]. In a study by Liu et al., the tunnel's boundaries were conventionally delineated by the attachments of the inferior extensor retinaculum as opposed to the tendons of the EHL and EDL [5]. A few anatomical studies in the existing literature have offered valuable insights into the ATT located amidst these tendons and the relationships between its contents [3,9]. However, there is a lack of studies from India, and the present study aims to provide a thorough depiction of the ATT in Indian cadavers and contribute anatomical knowledge to the existing literature.

The preliminary analysis of this study was presented at the Third International e-Conference "BMSeCON 2023," held in December 2023 in Pondicherry, India.

## Materials and methods

During the academic year 2023-2024, a descriptive study was carried out in the Department of Anatomy using 20 lower limbs from 10 formalin-fixed cadavers (male: 9; female: 1). These cadavers were procured by the institution through the Voluntary Body Donation Program with informed consent, written and signed by the donors themselves. The cadavers were approved for medical education and research in the Department of Anatomy. Resource constraints necessitated the adoption of a consecutive sampling technique, with the cadavers available in the department during the study period. Limbs exhibiting any indication of pathology, trauma, or surgical intervention were excluded from the study.

**Figure 1:**
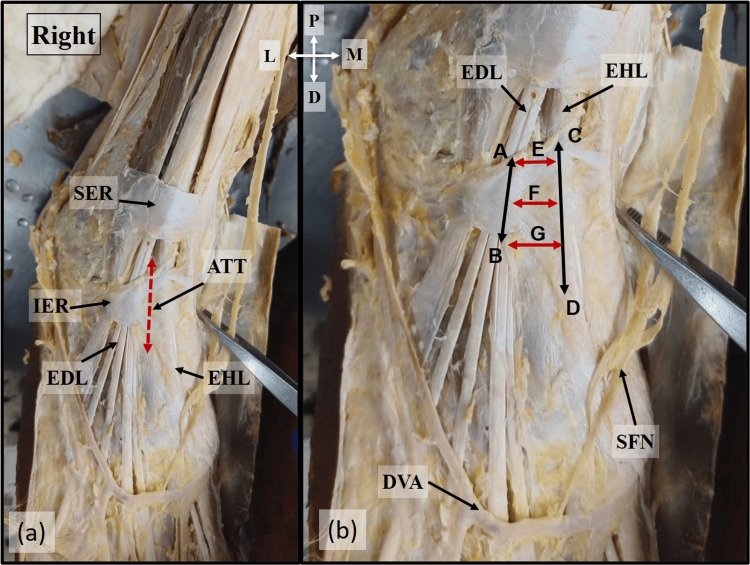
Location of ATT Location of the ATT in (a) and the dimensions of the ATT in (b). A-B: Lateral boundary. C-D: Medial boundary. E, F, and G denote the width of ATT at the proximal, middle, and distal ends ATT: anterior tarsal tunnel; EHL: extensor hallucis longus; EDL: extensor digitorum longus; SER: superior extensor retinaculum; DVA: dorsal venous arch; SFN: superficial fibular nerve; P: proximal; D: distal; M: medial; L: lateral

The dorsum of the foot was dissected following Cunningham's Manual of Practical Anatomy [10]. A horizontal skin incision was made from the lateral to the medial malleolus, and the incision was extended upwards and downwards from the midpoint of the horizontal incision. After reflecting the skin and removing the superficial fascia, the attachment of the inferior extensor retinaculum and its borders were defined. The tunnel was identified between the medial and lateral boundaries formed by the EHL and EDL tendons, respectively. The length of the tunnel extending between the superior and inferior borders of the inferior extensor retinaculum was measured at the medial boundary (EHL) and lateral boundary (EDL). The width of the tunnel was measured between the tendons of EHL and EDL at the proximal end, middle, and distal end of the tunnel (Figure 1). The dimensions of the tunnel were measured using Vernier callipers to the nearest millimetres.

Dissection was continued, and the inferior extensor retinaculum was cut between the two tendons to expose the tunnel. The contents of the tunnel, the DFN and DPA, were identified, and the relation between the DPA and DFN was analyzed. The branching pattern of the medial and lateral terminal branches of DFN within the tunnel was noted. The results were tabulated, statistical analysis was done using IBM SPSS Statistics for Windows, Version 20.0 (Released 2011; IBM Corp., Armonk, New York, United States), and a p-value of ≤0.05 was considered significant.

## Results

**Figure 2:**
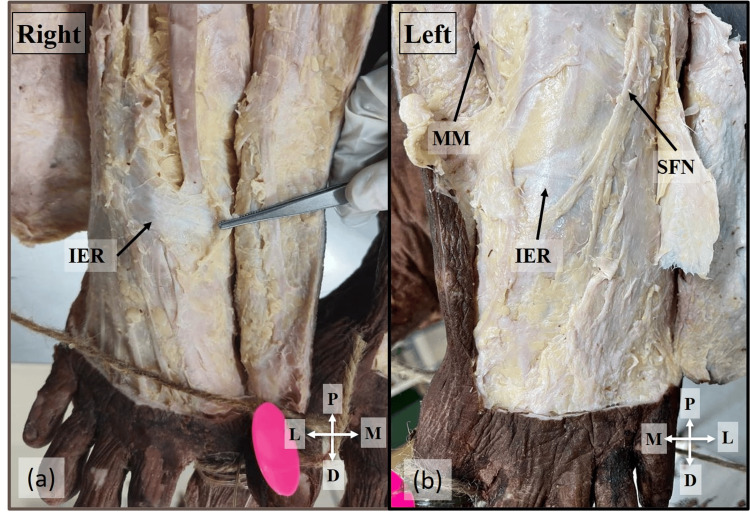
Absence of ATT Absence of the ATT due to the displaced IER distally from its normal position on the right (a) and left side (b) of the 60-year-old male cadaver ATT: anterior tarsal tunnel; IER: inferior extensor retinaculum; MM: medial malleolus; SFN: superficial fibular nerve; P: proximal; D: distal; M: medial; L: lateral

The mean length of the medial boundary was found to be 31.42±8.44 mm, while the lateral boundary was 20.39±4.39 mm. The mean width of the tunnel at the proximal end, middle, and distal end was 5.66±1.94 mm, 5.68±2.09 mm, and 9.18±2.17 mm, respectively. A comparison of the length of the tunnel between the right and left sides revealed a statistically significant difference, which is presented in Table 1. The above measurements showed that the tunnel widened from the proximal to the distal end. An interesting variation was observed in a 60-year-old male cadaver bilaterally, where the inferior extensor retinaculum was displaced distally on the dorsum of the foot, and a typical ATT was not observed. Instead, a single band was seen 47.25 mm away from the medial malleolus (Figure 2). The DFN coursed lateral to the DPA and bifurcated into terminal branches at the level where the distal end of the tunnel would have been located.

**Table 1:** Dimensions of the ATT in the present study ATT: anterior tarsal tunnel

Dimension	Mean value (mm)	Right side (mm)	Left side (mm)	P-value
Length				
Medial boundary	31.42±8.44	33.99±8.09	28.85±8.17	0.052
Lateral boundary	20.39±4.39	22.31±3.42	18.46±4.59	0.0047
Width				
Proximal end of the tunnel	5.66±1.94	5.65±1.29	5.675±2.47	0.97
Middle of the tunnel	5.68±2.09	6.13±1.81	5.22±2.29	0.17
Distal end of the tunnel	9.18±2.17	9.71±1.46	8.65±2.70	0.13

**Figure 3:**
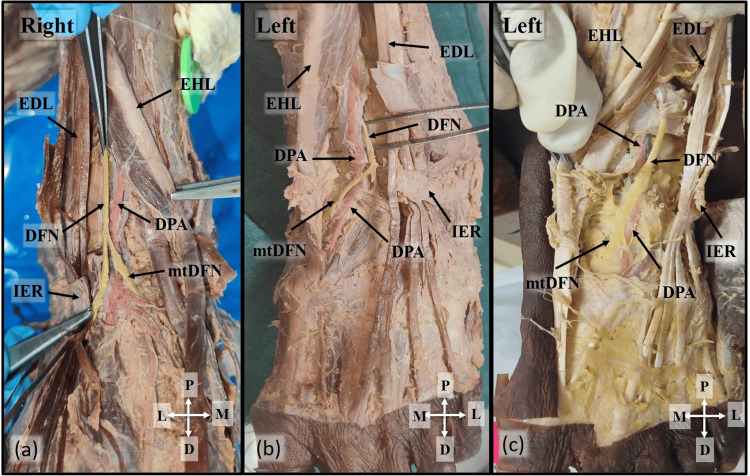
Contents of ATT IER is reflected to show the contents of the ATT. (a) The DFN is located lateral to the DPA and bifurcated proximal to the ATT. (b) DFN bifurcates within the ATT. (c) DFN crosses the DPA from the lateral to the medial aspect and bifurcates into its terminal branches at the distal part of the ATT IER: inferior extensor retinaculum; ATT: anterior tarsal tunnel; DFN: deep fibular nerve; DPA: dorsalis pedis artery; EHL: extensor hallucis longus; EDL: extensor digitorum longus; mtDFN: medial terminal branch of DFN; P: proximal; D: distal; M: medial; L: lateral

In 15 limbs, including the female cadaver, the DFN was located lateral to the DPA, while in five limbs, it was positioned medial to the DPA. The DFN branched into medial and lateral terminal branches within the tunnel in 11 limbs, proximal to the tunnel in one limb, and distal to the tunnel in six limbs (Figure 3).

## Discussion

A few anatomical studies have validated the presence of the ATT, although a comprehensive anatomical description is currently lacking in the literature [3,5,11]. Prior case reports and studies have used ultrasonograms, MRIs, and X-rays to examine the ATT and identify the cause of the symptoms resulting from the compression of the DFN [12,13]. In the present study, we have utilized cadavers to investigate the ATT between the tendons of EHL and EDL in the dorsum of the foot, the roof of which was formed by a part of the inferior extensor retinaculum stretching between the two tendons. Although the literature on the anatomy of the ATT is limited, a comparison of existing studies is presented in Table 2.

**Table 2:** Comparison of dimensions of the ATT with previous studies in the literature ATT: anterior tarsal tunnel

Study	Length		Width		
	Lateral boundary	Medial boundary	Proximal end	Middle	Distal end
Liu et al. 1991 [5]	15.7 mm	55.3 mm	-	-	-
Ikiz et al. 2007 [3]	21.7±4.2 mm	55.0±9.0 mm	-	4.7±2.4	12.6±2.1 mm
Present study 2024	20.39±4.39 mm	31.42±8.44 mm	5.66±1.94 mm	5.68±2.09 mm	9.18±2.17 mm

Compression of the DFN under the inferior extensor retinaculum was identified as a syndrome by Kopell and Thompson [3,14] and later named the "anterior tarsal tunnel syndrome" by Marinacci [15]. In China, Liu et al. demonstrated the existence of the tunnel anatomically using micro-dissection of specimens from 25 Chinese adults. Additionally, they provided clinical case reports that confirmed the nerve compression occurring within the tunnel [5]. A different case study employed roentgenograms to diagnose ATT syndrome in a patient experiencing numbness radiating to the first web space and pain in the dorsum of the foot. Osteophytes were identified on the dorsum of the talus, leading to subsequent surgical decompression of the ATT [12].

Liu et al. observed the length of the lateral boundary of the tunnel to be 15.7 mm [5], whereas Ikiz et al. reported a longer measurement of 21.7±4.2 mm [3]. Both studies showed comparable lengths of the medial boundary, as shown in Table 2. The length of the lateral boundary in the present study was similar to that of Ikiz et al. [3]. However, the medial border was shorter at 31.42±8.44 mm. Ikiz et al. observed that the width of the tunnel varied along its length, from the proximal to the distal end of the tunnel. They found that the width of the tunnel in the middle was narrow, measuring 4.7±2.4 mm, whereas, at the distal end, the width was 12.6±2.1 mm [3]. Similarly, in the present study, the width at the proximal end was 5.66±1.94 mm, which increased to 9.18±2.17 mm at the distal end of the tunnel.

The contents of the tunnel, the DFN, and the DPA had a variable relationship in the present study. The DFN was positioned lateral to the DPA in 15 limbs and medial in five limbs. Ikiz et al. conducted a study on 36 cadaveric lower limbs to investigate the relationship between the DFN and the DPA. Their findings revealed that in 22 limbs, the DFN was positioned lateral to the DPA, while in 11 limbs, the DFN and DPA crossed over each other at multiple levels [16]. In a similar study by Rab et al. involving 28 cadaveric lower limbs, the DFN was located laterally in 26 limbs and medially in two limbs, comparable to the present study [17]. A study from India reported that DFN and DPA crossed each other multiple times in 26.7% of cases, and in the rest, the DFN was lateral to the DPA [9]. In contrast, a study conducted by Turbpaiboon et al., encompassing 160 limbs, demonstrated that the DFN was laterally oriented in 79 limbs and medially oriented in 76 limbs [11]. The pattern of bifurcation of DFN in the present study is comparable to the study conducted by Ikiz et al., wherein an examination of 36 limbs revealed bifurcation of DFN within the tunnel in 31 limbs. In two limbs, the bifurcation occurred distal to the tunnel, and no bifurcations were observed in three specimens, which was attributed to the absence of the medial branch [16].

Stone et al. reported a case of ATT syndrome in a 47-year-old female, which occurred as a complication of ankle fusion with an anterior plate. The compression of DFN in the tunnel was caused by the anterior plate, which was subsequently managed through surgical intervention [18]. Compression of DFN by a thrombosed DPA leading to ATT syndrome has also been reported [6]. Repetitive plantarflexed and inverted foot in dancers makes the DFN susceptible to compression within the tunnel [19]. The other causes for ATT syndrome, as reported by Liu et al., were tight-laced footwear, idiopathic pes cavus, talonavicular osteophytes, and ganglia in ATT. The symptoms, such as continuous paresthesia and pain, were aggravated by high heels due to plantar flexion [5]. Akyüz et al. reported DFN entrapment neuropathy in 14 patients due to prolonged stretching of the DFN during prayer/namaz, which requires kneeling with the plantarflexed foot [20].

According to Takao et al., during ankle arthroscopic procedures, there is a high risk of injury to the contents of the tunnel when using the anterocentral portal [21]. Buckingham et al., in a study on cadaveric specimens, observed that the arthroscope contacted the DPA consistently in 90% of the cases and DFN was lacerated in one case when the anterocentral portal was used [8]. Similarly, in a cadaveric study on 10 ankle specimens by Inoue et al., the mean distance between the anterior neurovascular structures and the anterocentral portal was 2.2±2.0 mm, whereas the anteromedial and medial midline portals were 18.2±3.0 mm and 10.5±2.4 mm, respectively. The anterior neurovascular structures were in contact with the anterocentral portal in four specimens of the 10 specimens dissected [22].

## Conclusions

The present study explored the anatomy of the ATT, its morphometry, and its contents. Understanding this anatomy is crucial for analyzing ATT syndrome. A cautious approach is necessary to minimize the risk of neurovascular damage during ankle surgeries, arthroscopy, or injections in the ankle region. Awareness of the anatomy of ATT can lead to more effective treatment of ATT syndrome rather than misattributing it to lumbar radiculopathy. Consequently, this study contributes to existing literature and paves the way for future research, despite limitations such as a small sample size and a limited number of female cadavers.
